# Identification, characterization, and validation of NBS-encoding genes in grass pea

**DOI:** 10.3389/fgene.2023.1187597

**Published:** 2023-06-20

**Authors:** Alsamman M. Alsamman, Khaled H. Mousa, Ahmed E. Nassar, Mostafa M. Faheem, Khaled H. Radwan, Monica H. Adly, Ahmed Hussein, Tawffiq Istanbuli, Morad M. Mokhtar, Tamer Ahmed Elakkad, Zakaria Kehel, Aladdin Hamwieh, Mohamed Abdelsattar, Achraf El Allali

**Affiliations:** ^1^ Agricultural Genetic Engineering Research Institute (AGERI), Agricultural Research Center (ARC), Giza, Egypt; ^2^ International Center for Agricultural Research in the Dry Areas (ICARDA), Giza, Egypt; ^3^ International Center for Agricultural Research in the Dry Areas (ICARDA), Terbol, Lebanon; ^4^ African Genome Center, Mohammed VI Polytechnic University, Ben Guerir, Morocco; ^5^ Department of Genetics and Genetic Engineering, Faculty of Agriculture at Moshtohor, Benha University, Benha, Egypt; ^6^ Moshtohor Research Park, Molecular Biology Lab, Benha University, Benha, Egypt; ^7^ Biodiversity and Crop Improvement Program, International Center for Agricultural Research in the Dry Areas (ICARDA), Rabat, Morocco

**Keywords:** NBS, grass pea (Lathyrus sativus L), gene expression, genome wide analysis, real time—PCR, legume, biotic stress, abiotic stress

## Abstract

Grass pea is a promising crop with the potential to provide food and fodder, but its genomics has not been adequately explored. Identifying genes for desirable traits, such as drought tolerance and disease resistance, is critical for improving the plant. Grass pea currently lacks known R-genes, including the nucleotide-binding site-leucine-rich repeat (NBS-LRR) gene family, which plays a key role in protecting the plant from biotic and abiotic stresses. In our study, we used the recently published grass pea genome and available transcriptomic data to identify 274 NBS-LRR genes. The evolutionary relationships between the classified genes on the reported plants and LsNBS revealed that 124 genes have TNL domains, while 150 genes have CNL domains. All genes contained exons, ranging from 1 to 7. Ten conserved motifs with lengths ranging from 16 to 30 amino acids were identified. We found TIR-domain-containing genes in 132 LsNBSs, with 63 TIR-1 and 69 TIR-2, and RX-CCLike in 84 LsNBSs. We also identified several popular motifs, including P-loop, Uup, kinase-GTPase, ABC, ChvD, CDC6, Rnase_H, Smc, CDC48, and SpoVK. According to the gene enrichment analysis, the identified genes undergo several biological processes such as plant defense, innate immunity, hydrolase activity, and DNA binding. In the upstream regions, 103 transcription factors were identified that govern the transcription of nearby genes affecting the plant excretion of salicylic acid, methyl jasmonate, ethylene, and abscisic acid. According to RNA-Seq expression analysis, 85% of the encoded genes have high expression levels. Nine LsNBS genes were selected for qPCR under salt stress conditions. The majority of the genes showed upregulation at 50 and 200 *μ*M NaCl. However, *LsNBS-D18*, *LsNBS-D204*, and *LsNBS-D180* showed reduced or drastic downregulation compared to their respective expression levels, providing further insights into the potential functions of LsNBSs under salt stress conditions. They provide valuable insights into the potential functions of LsNBSs under salt stress conditions. Our findings also shed light on the evolution and classification of NBS-LRR genes in legumes, highlighting the potential of grass pea. Further research could focus on the functional analysis of these genes, and their potential use in breeding programs to improve the salinity, drought, and disease resistance of this important crop.

## 1 Introduction

The grass pea (*Lathyrus sativus*) is a crucial legume for human sustenance due to its drought resistance and high yield properties (Yan et al., 2006; Hillocks and Maruthi, 2012).It is an ideal choice for sustainable food production in arid regions, as it requires little water, has high a protein content, and is drought-tolerant (Campbell et al., 1994; Dixit et al., 2016). In addition to its nutritional benefits, grass pea is an excellent source of L-homoarginine (Har), an uncommon non-protein amino acid that is a substrate for regulated nitric oxide production and is essential for the treatment of cardiovascular disorders (Das et al., 2021). Moreover, as a nitrogen-fixing plant, it plays a vital role in the agricultural system by providing a nitrogen source for subsequent crops (Skiba et al., 2007). Grass pea belongs to the Fabaceae family, with a diploid chromosome number of 14 (2n = 14) and a genome size of 8.12 GB (Almeida et al., 2015; Alsamman et al., 2023). It is one of the 150 species in the genus Lathyrus, found in various tropical and subtropical climates worldwide, including Iraq, Iran, Afghanistan, Syria, India, Pakistan, Morocco, and temperate regions of South America (Girma and Korbu, 2012). Considering the nutritional potential and adaptability of grass pea, there is a pressing need to intensify research efforts to improve our understanding of this plant, particularly given the current economic conditions.

Plants grown in challenging environments, such as the grass pea, are subjected to a range of diseases and environmental stressors throughout their development. Due to a relatively weak immune system and exposure to changing environmental conditions, diseases like powdery mildew (*Erysiphe pisi*), downy mildew (*Peronospora lathyrus*), rust (*Uromyces pisi*), and parasitic weed (*Orobanche crenata*) can have severe consequences on crop yield (Fernández-Aparicio et al., 2016; Infantino et al., 2016). Powdery mildew, for example, leads to significant reductions in photosynthetic activity and physiological changes that can result in yield losses of 20%–40%, depending on the timing and severity of the disease (Kahate and Kahate, 2022). To defend against these pathogens, plants have developed a complex innate immune system comprising multiple layers of defensive proteins. Effector-Triggered Immunity (ETI) is one such layer that functions within the cell via proteins encoded by the R gene family of defense genes (Lozano et al., 2015). Induction of disease resistance by the R gene is among the most critical plant defense mechanisms (Dangl and Jones, 2001).

Plant resistance to diseases is primarily governed by a set of genes called R genes that have been extensively studied in plants (Kourelis and Van Der Hoorn, 2018; Mokhtar et al., 2023a). Most R genes belong to the nucleotide-binding site leucine-rich repeat (NBS-LRR) gene family, characterized by the presence of nucleotide binding site (NBS) and leucine-rich repeat (LRR) domains (McHale et al., 2006). The NBS-LRR genes function primarily as intracellular receptors and recognize the presence of pathogen effectors through direct binding to pathogen effector proteins or indirectly by detecting any changes in pathogen effector target proteins in the host, such as Avr proteins (Chisholm et al., 2006; Sagi et al., 2017). The NBS-LRR genes can be divided into two subfamilies; TIR-NB-LRR (TNL) proteins that contain Toll/interleukin-1 receptor (TIR) domains, and CC-NB-LRR (CNL) proteins that contain coiled-coil (CC) domains (Afrin et al., 2018). These proteins are frequently utilized as pathogen sensors, and when the LRR domain binds to pathogen effectors, the TNL or CNL protein undergoes a conformational shift, causing the TIR or CC domain to multimerize and activate the immune system (Andersen et al., 2018; Kourelis and Van Der Hoorn, 2018). Hundreds of NBS-LRR genes have been found in the genomes of maize, apple, sweet potato, Arabidopsis, and chickpea due to their essential role in plant immunology and abundance in plant genomes. However, no systematic investigation of NBS-LRR genes in grass pea has been published (Huang et al., 2020).

In this study, our primary objective was to identify the complete set of NBS-LRR genes in the grass pea genome and explore their functionality and evolutionary path through various bioinformatics and laboratory techniques. To achieve this, we identified and classified the NBS-LRR genes into TNL and CNL subfamilies and then studied their motifs, domains, and annotations to assess their viability and functionality. Furthermore, we analysed potential functions, gene interactions, and transcription factors. We used published RNA-seq data to evaluate their expression under different conditions, particularly pathogen infections. This approach allowed us to identify genes with the highest levels of expression, and we selected nine of these genes for validation using quantitative real-time PCR.

## 2 Materials and methods

### 2.1 Genomic data

The genomic data for the grass pea genotype LS007 was retrieved from the NCBI database (https://www.ncbi.nlm.nih.gov, NCBI ID: CABITX010000000). The genome assembly had a size of 8.12 Gbp, a coverage of 60X, an N50 of 59,728 bp, and a total of 669,893 sequences, as previously reported (Emmrich et al., 2020). In addition, transcriptomic sequences totaling 103.3 Mbp were obtained from NCBI (https://www.ncbi.nlm.nih.gov/bioproject/PRJNA258356) to augment the genomic data. NBS-LRR protein sequences from previously published studies, including those from chickpea Sharma et al. (2017), apple Arya et al. (2014), and *Brassica napus* Alamery (2015), were used as sequence models for gene identification and classification.

### 2.2 Identification of NBS-LRR genes in grass pea genome

To identify potential loci of NBS-LRR genes across the Lathyrus genome using the NBS-LRR genes retrieved from other plant species, we used Local TBLASTN with a sequence similarity threshold of 90% and a sequence length of 600 nucleotides. To predict possible coding regions, we used TransDecoder (Release v5.5.0) (Thunders et al., 2017). Protein sequences obtained from TransDecoder were screened for domains using “hmmsearch” (Johnson et al., 2010) (v3.1b2; http://hmmer.org), which used a Hidden Markov Model (HMM) with the NBS domain (pfam00931) retrived from the Pfam database (Mistry et al., 2021). We subjected potential LsNBS genes to the NCBI-CDD tool for conserved domain verification (Marchler-Bauer et al., 2010; Yang et al., 2020). To evaluate the gene structure of the identified sequences and predict alternative transcripts, we used the AUGUSTUS tool (version 3.3) (Hoff and Stanke, 2019).

### 2.3 Gene family classification using phylogenetic analysis

The potential LsNBS genes were aligned with previously classified genes from chickpea, apple, and Brassica napus using MUSCLE (Multiple Sequence Comparison by Log-Expectation) (v3.8.1551; https://www.drive5.com/muscle/) (Edgar, 2004) to identify NBS-LRR subfamilies, including TIR-domain-containing (TNL) and CC-domain-containing (CC/CNL) subfamilies. The evolutionary relationships of the LsNBS genes were evaluated using RAxML (Randomized Axelerated Maximum Likelihood) algorithm (Stamatakis, 2014) with an alignment model of ”PROTGAMMAWAG”, and the best bootstrap was chosen randomly. The potential tree was chosen from ten different phylogenetic trees generated in the process. The analysis was performed on a supercomputer using 56 cores in a multi-threading process. The resulting phylogenetic tree was visualized using the online tool iTOL (https://itol.embl.de/) (Letunic and Bork, 2021).

### 2.4 Conserved motifs and gene structure analysis

Conserved motifs in the potential LsNBS proteins were identified using the MEME-Suite tool (Bailey et al., 2015) (https://meme-suite.org/meme/tools/meme), with optimum motif width ranges from 6 to 50 bp and a maximum number of motifs of 10. The Gene Structure Display Server (GSDS) was used to display motif structure, resolved domains, intron-exon structure, and evolutionary relationships of requested genes generated using the sequence alignment analysis.

### 2.5 Expression pattern of LsNBS based on RNA-seq data

To validate gene expression patterns of the identified NBS-LRR genes, we retrieved RNA-seq data from previously published datasets in the NCBI Sequence Read Archive (NCBI-SRA/https://www.ncbi.nlm.nih.gov/sra) database. Four experiments (SRP045652, SRP092875, SRP145030, and SRP327502) were retrieved from the NCBI-SRA database. The RNA-seq data from these experiments were used to validate the expression of the identified NBS-LRR and to determine their levels of expression in different plant tissues and under different stress conditions. Providing further details on these studies, SRP145030 was part of a transcriptomic investigation into the germination of grass peas over 2, 6, and 25 days (Xu et al., 2018). Meanwhile, SRP045652 explored gene expression in seedlings that were subjected to stress with 2 mg of spores per plant. SRP092875 (Hao et al., 2017) evaluated six samples taken from root, stem, and leaf tissues. SRP327502 focused on the transcriptome profiling of grass pea accessions with contrasting Beta-ODAP content and was isolated from seedling shoot tissues at 4 and 7 days post-germination. It is worth noting that SRP327502’s main objective was to compare accessions with differing Beta-ODAP contents. Overall, these studies provide valuable insights into gene expression and transcriptomic changes during plant growth and stress responses. A sample quality control approach was implemented using FASTQC (de Sena Brandine and Smith, 2019). To remove low-quality sequences, the fqtrim (Diroma et al., 2019) and fastq_quality_filter (Fu and Dong, 2018) tools were utilized. Hisat2 Vandenbrink et al. (2019) was then deployed to map the sequenced reads to the LsNBS genes, while Htseq (Anders et al., 2015) was used to count the aligned reads based on the gene annotation. These tools were chosen based on their ability to accurately and efficiently process large volumes of sequencing data while maintaining high-quality standards. By utilizing these tools in our analysis, we can ensure that the sequencing data is of high quality, accurately mapped to the reference genome, and provides reliable and informative gene expression data.

### 2.6 Gene enrichment, and *cis*-elements analyses

We used the STRING (Szklarczyk et al., 2021) online database to identify protein-protein interactions (PPIs) among the identified LsNBSs, Using the *Cicer arietinum* as an annotation model. The resulting PPI network was visualized with Cytoscape software (Kohl et al., 2011), where nodes represent proteins and edges represent known interactions. We then downloaded the functional enrichment of LsNBS genes from the STRING database and visualized it using the R ggplot2 package. To identify *cis*-elements, we extracted the 1.5 kb upstream sequences from the promoter regions of the LsNBS genes and used PlantCARE (Lescot et al., 2002) to identify CREs. Finally, we displayed the *cis*-element areas of each gene using Tbtools (Chen et al., 2020).

### 2.7 Plant materials, growth conditions, and stress treatment

The experimental material used in this study consisted of grass pea seeds obtained from the International Center for Agricultural Research in the Dry Areas (ICARDA) genebank. The seeds were germinated in a 3:1:1 (v/v/v) mixture of nutrient soil, vermiculite, and perlite and grown in pots. The plants were then placed in a controlled greenhouse with a photoperiod of 16/8 h (day/night), 80% relative humidity, and a temperature of 25°C/18°C (day/night). Photosynthetically active radiation of 250 mol *m* ^2^ *s* ^−1^ was supplied by cool white fluorescent lamps. Salinity treatment was applied to the plants 30 days after sowing by adding sodium chloride (NaCl) to the soil at concentrations of 0, 50, 100, and 200 µM Three biological replicates were collected 72 h post-treatment, consisting of one leaf from each seedling, flash frozen immediately, and stored at 80°C.

### 2.8 RNA extraction and RT-qPCR analysis

Total RNA was extracted from both control and stressed grass pea leaves using the TRIzol reagent (Invitrogen, Carlsbad, CA, United States) according to the manufacturer’s instructions. To remove genomic DNA contamination, RNA samples were treated with RNase-free DNase (Promega Corporation, Madison, WI, United States). The quality and quantity of the extracted RNA were assessed using a NanoDrop^™^ 2000 spectrophotometer (Thermo Fisher Scientific, Waltham, MA, United States), and 1.5 *μg* of RNA was used for cDNA synthesis. The M-MLV Reverse Transcription Kit (Promega Corporation, United States) was used to synthesize cDNA, which was then diluted to 1/10 before quantitative PCR (qPCR) analysis. The qPCR-specific primers were designed using the PrimerQuest Tool (https://www.idtdna.com/PrimerQuest/Home/Index) (Table 1).

**TABLE 1:** qPCR Primer sequences for Real-Time PCR validation of identified LsNBSs, with Elf1b and ABCT genes as control/reference genes.

Gene	Forward primer sequence	Reverse primer sequence
LsNBS-R18	CCAGAAATCACTGGAAGAATAGA	CCTCCAATACCCACAATAGC
LsNBS-D88	ACCTGACAGAGTTGATACAAAG	GATCACTTCACGCAACAAATC
LsNBS-D142	CAGGGAATGTAGGAGTTTGG	CGTTGAGGTAGTGATCTAAGTG
LsNBS-D146	GTCTTCACAATGACGGAAATG	GGCAATCCTCCGCAATAA
LsNBS-D180	GATGTACGACCCAACACTATG	CCATTCCATGCAAACCAATTAT
LsNBS-D204	GTGCATTCAGCTATCGAACTA	CCCATTGAACCTAAGAGAAGG
LsNBS-R4	GATTCTCTAACGCTGTCTGATT	CTTTGGGCACCTGTCTATTT
LsNBS-R21	CTCGCTTCCCTTGAAGTATTG	GTCTGAGCTGTAGCCTCTTA
LsNBS-R98	GTTTCCTACACTTAGCACATTTC	CCTTCTGCCACCCATATTC
Ls_Elf1b	ATCCCTCAACGATTTCCT	AGACACAGCATCATACCA
Ls_ABCT	GGCATTTCCCTATGTTTCAG	TCACCTTCAGATTGTGTTCT

The expression level of the selected nine LsNBSs in all samples was assessed through qPCR analysis using the BioEasy Master Mix Plus (SYBR Green Mix; BIOER, Hangzhou, China) on an Agilent Stratagene Mx3005p real-time PCR detection system, following the manufacturer’s instructions. For expression analysis, a total volume of 20 *μ* l was used, comprising 1 *μ*L of diluted cDNA, 10*μ*L of 2 × SYBR Green PCR Master Mix, and 0.4 *μ*L of each forward and reverse primer (10 *μ*m). Amplification was performed using the following program: 95°C for 60 s, followed by 40 cycles at 95°C for 3 s and 60°C for 40 s. To ensure the specificity of qPCR products, a melting curve analysis was conducted from 65°C to 95°C. Normalization was done using internal reference genes, *ABCT* and *Elf1b*-specific primers (Zhang Y. et al., 2022). The relative expression levels of all reactions, run in triplicate, were calculated using the 2^−△△^^*Ct*^ method (Schmittgen and Livak, 2008).

## 3 Results

### 3.1 Gene identification and phylogenetic analysis

**FIGURE 1:**
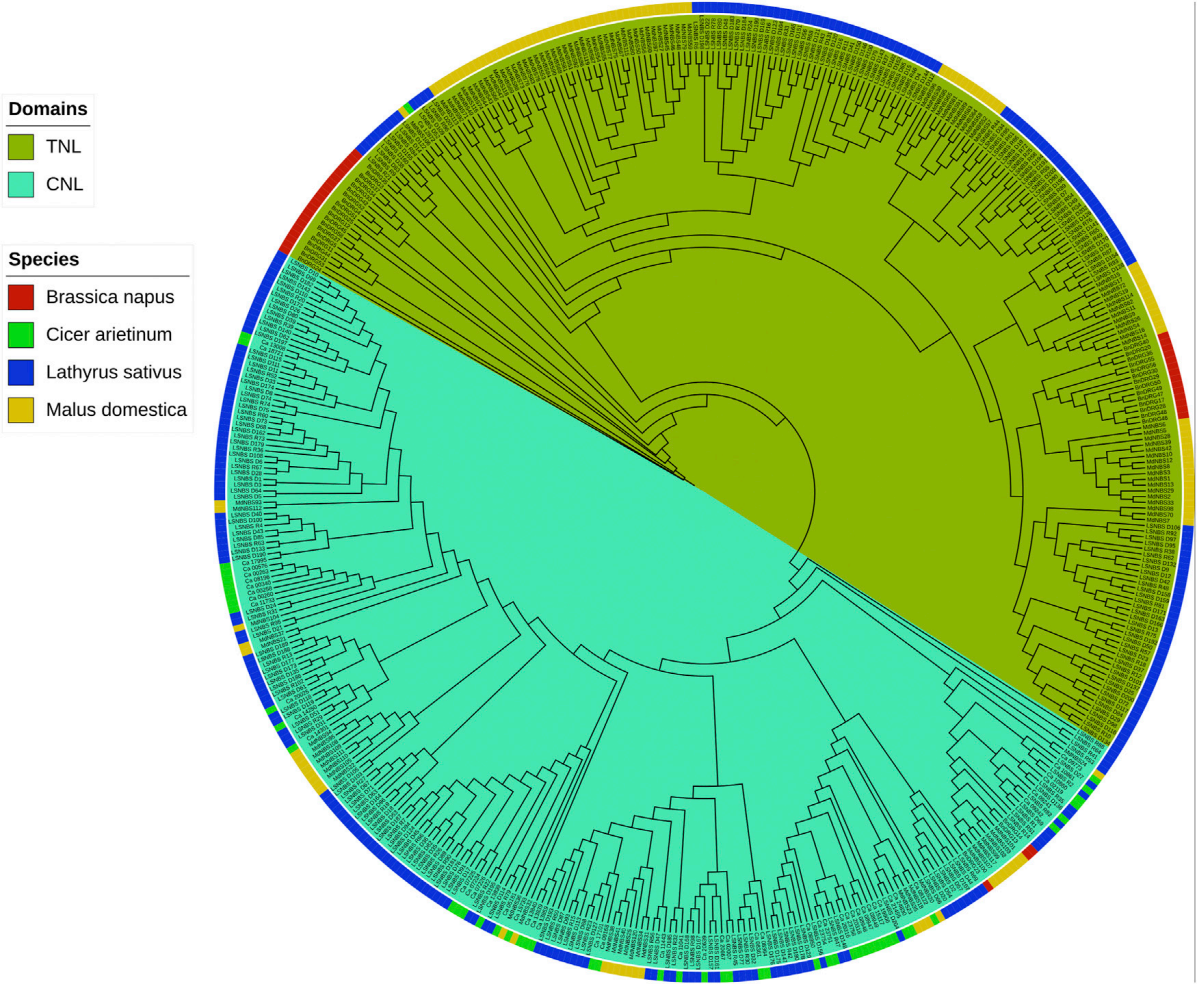
Phylogenetic relationships of NBS domain-containing genes (CNL and TNL) in grass pea, chickpea, apple, and Brassica napus inferred using the Maximum Likelihood method. The resulting phylogenetic tree divided the 274 LsNBS genes into two subfamilies: the TNL subfamily, which contains 124 LsNBS genes, and the CNL subfamily, which contains the other 150 LsNBS genes.

LRR genes were retrieved from other plant species as queries to identify potential genomic loci located on the grass pea genome and transcriptome, which led to the identification of 307 potential R genes. Among these sequences, 274 genes were annotated as complete genes, while 33 were excluded as non-functional genes (Supplementary Table S3). The length of amino acid sequences ranged from 86 aa (*LsNBS-D2*) to 1,505 aa (*LsNBS-R42*), with an average length of 571.66 aa (Supplementary Table S2). The molecular weight of these amino acids ranged from 172.3 kDa to 9.7 kDa, with an average weight of 65.5 kDa (Supplementary Table S5). The theoretical isoelectric point ranged from 10.1 in *LsNBS-R96* to 4.2 in *LsNBS-D119* (Supplementary Table S6). We constructed a phylogenetic tree with 209 TNL-CNL LsNBS containing proteins (37 BnDRG, 52 chickpea (capoli), and 120 MdNBS) to classify the identified LsNBS genes into subfamilies, the evolutionary relationships between the classified genes on the reported plants and LsNBS revealed that 124 genes have TNL domains, while 150 have CNL (Figure 1). The phylogenetic tree was separated into two clades, where the first comprises 245 genes with TNL domains, while the second has 238 genes with CNL domains. This classification approach serves as a crucial protocol in gene family analysis, enabling us to categorize genes based on their specific domains. It has been widely employed in numerous studies to gain a better understanding of the evolutionary relationships and functional diversity of gene families (Li et al., 2022). By utilizing this methodology, we obtained valuable insights into the classification and evolutionary dynamics of the LsNBS genes, particularly within the context of other plant species. This analysis contributes to our broader understanding of the evolutionary patterns and functional characteristics of gene families in plants.

We used known NBS-LRR genes retrieved from other plant species as queries to identify potential genomic loci located on the grass pea genome and transcriptome, which led to the identification of 307 potential R genes. Among these sequences, 274 genes were annotated as complete genes, while 33 were excluded as non-functional genes (Supplementary Table S2; Supplementary Table S3; Supplementary Table S4). The length of the amino acid sequences ranged from 86 aa (*LsNBS-D2*) to 1,505 aa (*LsNBS-R42*), with an average length of 571.66 aa (Supplementary Table S2). The molecular weight of these amino acids ranged from 9.7 kDa to 172.3 kDa, with an average weight of 65.5 kDa (Supplementary Table S5). The theoretical isoelectric point ranged from 4.2 in *LsNBS-D119* to 10.1 in *LsNBS-R96* (Supplementary Table S6). To classify the identified LsNBS genes into subfamilies, we constructed a phylogenetic tree with 209 TNL-CNL LsNBS-containing proteins (37 BnDRG, 52 chickpea, and 120 MdNBS). The evolutionary relationships between the classified genes on the reported plants and LsNBS revealed that 124 genes have TNL domains, while 150 have CNL domains. The phylogenetic tree was separated into two clades, with the first comprising 245 genes with TNL domains and the second comprising 238 genes with CNL domains (Figure 1).

### 3.2 Gene structure and conserved motifs analysis

**FIGURE 2:**
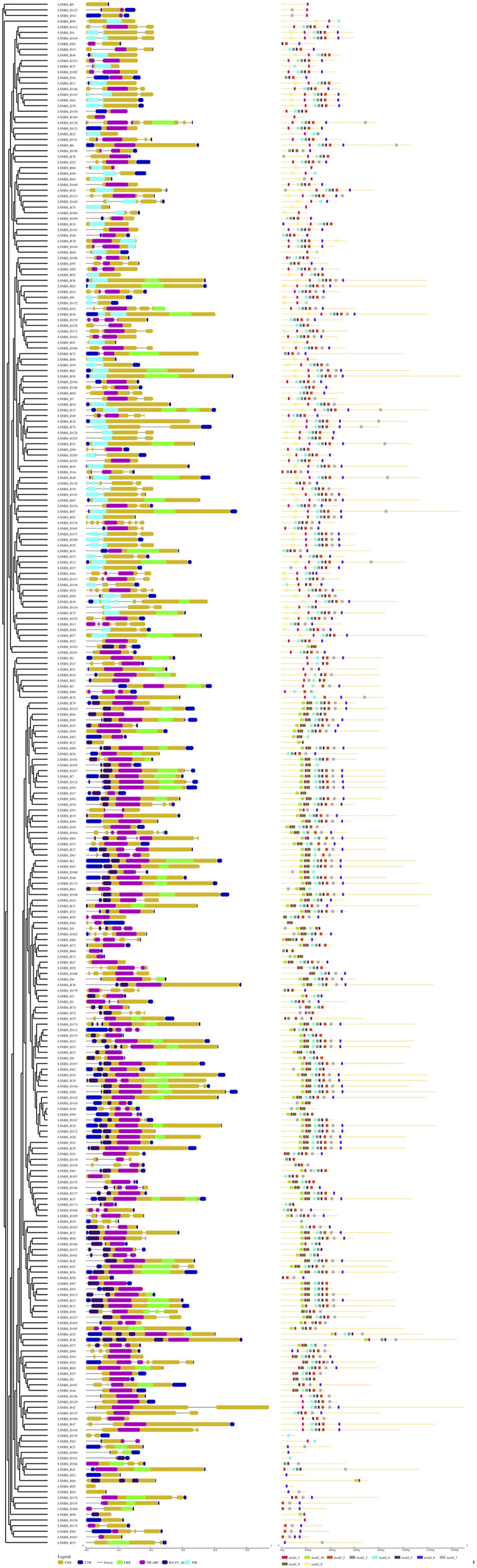
LsNBS protein gene structures (left), with introns indicated by black lines and the distribution of conserved motifs in LsNBS proteins depicted on (right).

The variation in exon-intron structure and distribution among gene family members is crucial for the emergence of diverse gene families and provides valuable evidence for their evolutionary relationships (Hu and Liu, 2012; Xu et al., 2012). In this study, we thoroughly examined the amino acid sequences of 274 LsNBS genes to identify motifs and protein domain structures. We found that all genes contain exons, with the number of exons ranging from 1 to 7. For instance, genes such as *LsNBS-D2*, *LsNBS-D14*, and *LsNBS-D16* have only one exon, while *LsNBS-D128* has seven exons. The intron distribution varied from 1 to 6, with genes such as *LsNBS-D3*, and *LsNBS-D6* having only one intron, while *LsNBS-D128*, and *LsNBS-D164* have six introns. However, no introns were identified in 43 and 55 LsNBS genes in the grass genome and transcriptome, respectively. We identified ten conserved motifs in the structure of the identified LsNBS genes (Figure 2), with motif lengths ranging from 16 to 30 amino acids. While all genes contain motifs, some, such as *LsNBS-R9*, *LsNBS-R100*, *LsNBS-D176*, and *LsNBS-D109*, have only one motif, while others, such as *LsNBS-R12* and *LsNBS-R10*, have eight motifs (Figure 2). We also identified several popular motifs, including P-loop, Uup, kinase-GTPase, ABC, ChvD, CDC6, Rnase_H, Smc, CDC48, and SpoVK. Moreover, we found TIR-domain-containing (TIR) genes in 132 LsNBSs, with 63 TIR-1 and 69 TIR-2, when NBS domains were examined. Additionally, RX-CC Like were found in 84 LsNBSs (File S7). These results provide important insights into the diversity and evolution of LsNBS genes.

### 3.3 Gene enrichment analysis and PPI network construction

**FIGURE 3:**
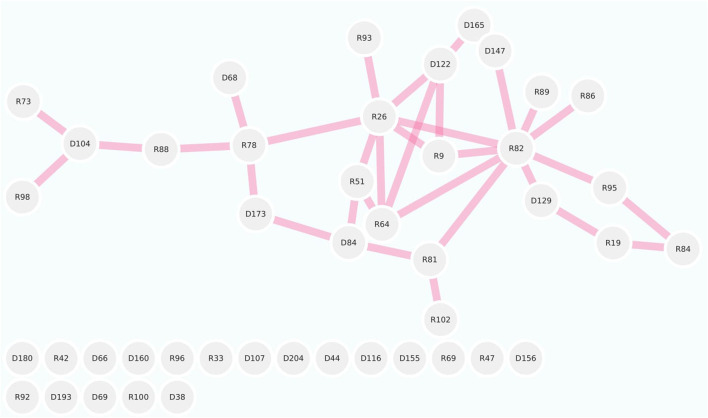
LsNBS protein-protein interaction network generated using STRING database using chickpea as plant model.

**FIGURE 4:**
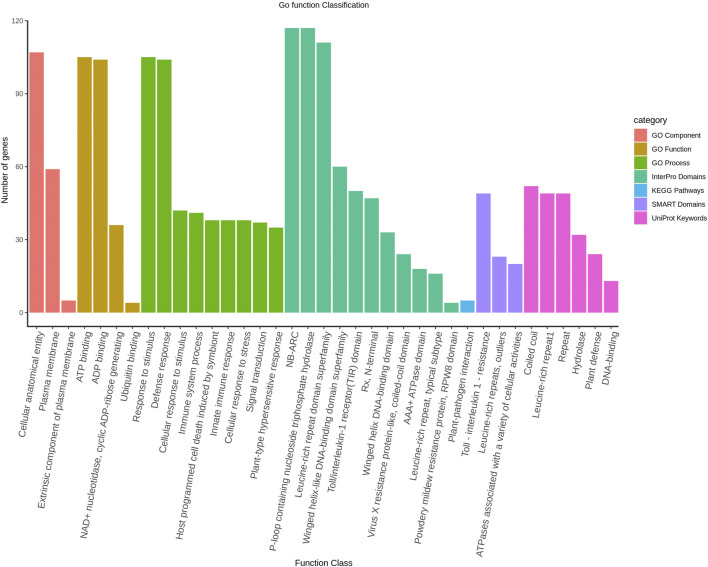
The Gene Ontology classification of LsNBS genes based on their involvement in Biological Process, Cellular Component, and Molecular Function with a significance level of FDR < 0.05.

The protein interaction among the LsNBS genes was investigated to determine potential activity and relationships. The STRING database annotated and categorized the 274 NBS-LRR domains into TIR-NBS-LRR (TIR) and CC-NBS-LRR (CNL) and found that the *LsNBS-R82*, *LsNBS-R26*, and *LsNBS-R9* genes were highly interactive and situated in the core of the interaction network (Figure 3). However, we noticed that several genes were not interactive, which could be due to the low amount of information available for these genes in the database or literature. The gene enrichment analysis revealed several noteworthy features and confirmed the functional analysis, thereby aiding in the understanding of their diverse molecular functions (Supplementary Table S9). Furthermore, some of these features were corroborated by different databases Ashburner et al. (2000). We identified 52 LsNBSs involved in plant innate immunity and defense, hydrolase, and DNA-binding, while 49 LsNBSs were implicated in Toll-interleukin-1 resistance, leucine-rich repeats, and outlier ATPases associated with various cellular activities. Additionally, 105 LsNBS genes were involved in response to stimulus, host programmed cell death, signal transduction, and the hypersensitive response. We classified 105 LsNBS genes as being involved in ATP binding, ADP binding, NAD + nucleotidase, cyclic ADP-ribose generating, and Ubiquitin binding, while 107 LsNBSs were categorized as being involved in a cellular anatomical entity, plasma membrane, and extrinsic component (Figure 4).

### 3.4 *Cis*-acting elements in the LsNBS promoters

**FIGURE 5:**
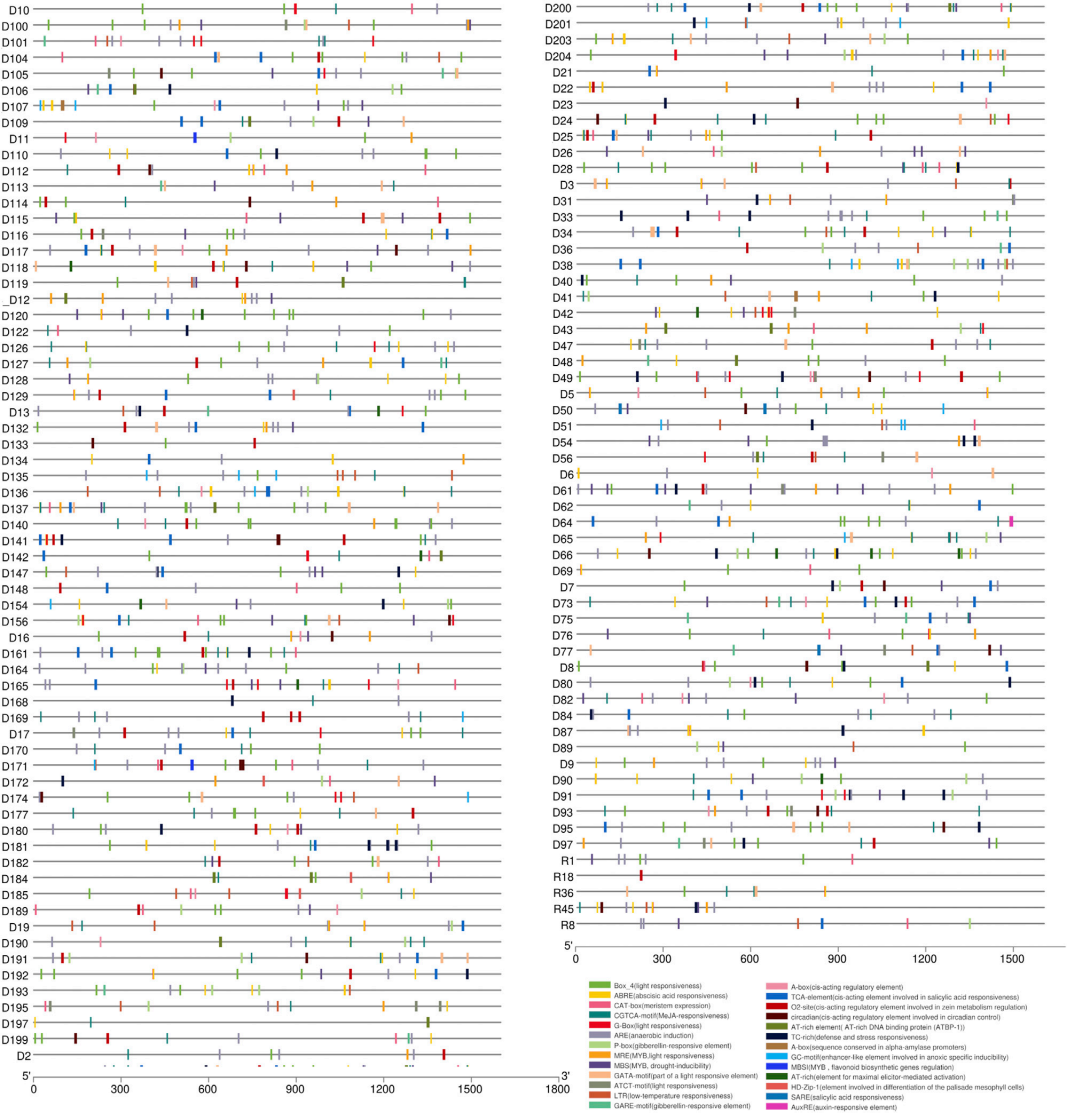
The putative *cis*-elements identified in the 1,500 bp upstream region of the LsNBS genes in grass pea, which are involved in plant hormone regulation and stress resistance.

*Cis*-elements are essential components of gene promoters and play a critical role in gene expression and function (Yin et al., 2020; Huang et al., 2021). In this study, we performed *cis*-element identification analysis to locate putative regulatory elements of the 274 reported LsNBSs by examining the 1.5 kb upstream region of each gene (Figure 5; Supplementary Table S8). Our analysis revealed that the CAAT box was a common binding component of all LsNBS genes, while the TATA box was identified in 72 LsNBS genes. Furthermore, various forms of *Cis*-elements (CREs) were found in 101 LsNBS genes. To investigate stress-specific promoter elements in NBS genes, we identified four types of hormone-responsive regulatory elements including, TCA (salicylic acid responsiveness), CGTCA/TGACG-motifs (Methyl Jasmonate responsiveness), Ethylene Responsive Element (ERE), and ABRE (abscisic acid responsiveness). In addition, regulatory regions contained several other motifs, including Box 4, G-Box, GT1-motif, AE-box, GATA-motif, TCT-motif, Sp1, AT1-motif, I-box, LAMP-element, TCT-motif, and chs-CMA1a, which modulate plant light response. The presence of TC-rich repeats in some of the LsNBS genes suggests their role in regulating plant defense and stress reactivity (Wei et al., 2009). Another key repeat that emerged in our analysis was the DRE repeat, which was found in 13 LsNBS upstream regions and is associated with stressful conditions such as dehydration, low temperature, and salt stressors (Meena et al., 2022). This finding is particularly significant for plants, such as grass pea, which are native to arid regions.

In our analysis of the diverse array of cis-acting elements, particularly those associated with stress conditions, it is worth highlighting the specific cis-elements that were frequently observed in the reported LsNBSs (Figure 5) and Supplementary Table S8. Among them, certain elements stood out with high occurrence: CCAAT (396 times), TGACG (256 times), ACGTG (144 times), CACGTG (63 times), and CCGAAA (42 instances). These cis-elements are known to function as binding sites that control the expression of crucial plant genes. The CCAAT motif is a well-studied cis-element that plays a crucial role in various aspects of plant biology, particularly in plant defense (Zhao H. et al., 2020). Members of the NF-Y transcription factor complex bind to the CCAAT box in gene promoters, influencing plant development and adaptation to abiotic stresses (Maheshwari et al., 2019). TGACG is a regulatory motif that significantly regulates tissue-specific gene expression and has potential for enhancing crop productivity through genetic manipulation of plant promoters (Kumar et al., 2012). ACGTG is a cis-element closely related to plant growth, development, signal transduction, and stress response. It, along with other cis-elements, can serve as valuable tools for studying genetic diversity, analyzing relationships between different species, and facilitating breeding efforts (Chen et al., 2022). CACGTG is a cis-element known for its importance in plant defense against pathogens. The G-box (CACGTG) cis-element functions in the activation of genes involved in the production of lignin precursors, phytoalexins, and salicylic acid, which are early responses to pathogen attack (Raikwar et al., 2015). The CCGAAA motif plays a significant role in regulating plant responses to cold stress and sugar signaling, ultimately influencing plant defense (Qian et al., 2018).

### 3.5 Expression patterns of NBS-LRR genes in *Lathyrus sativus*

**FIGURE 6:**
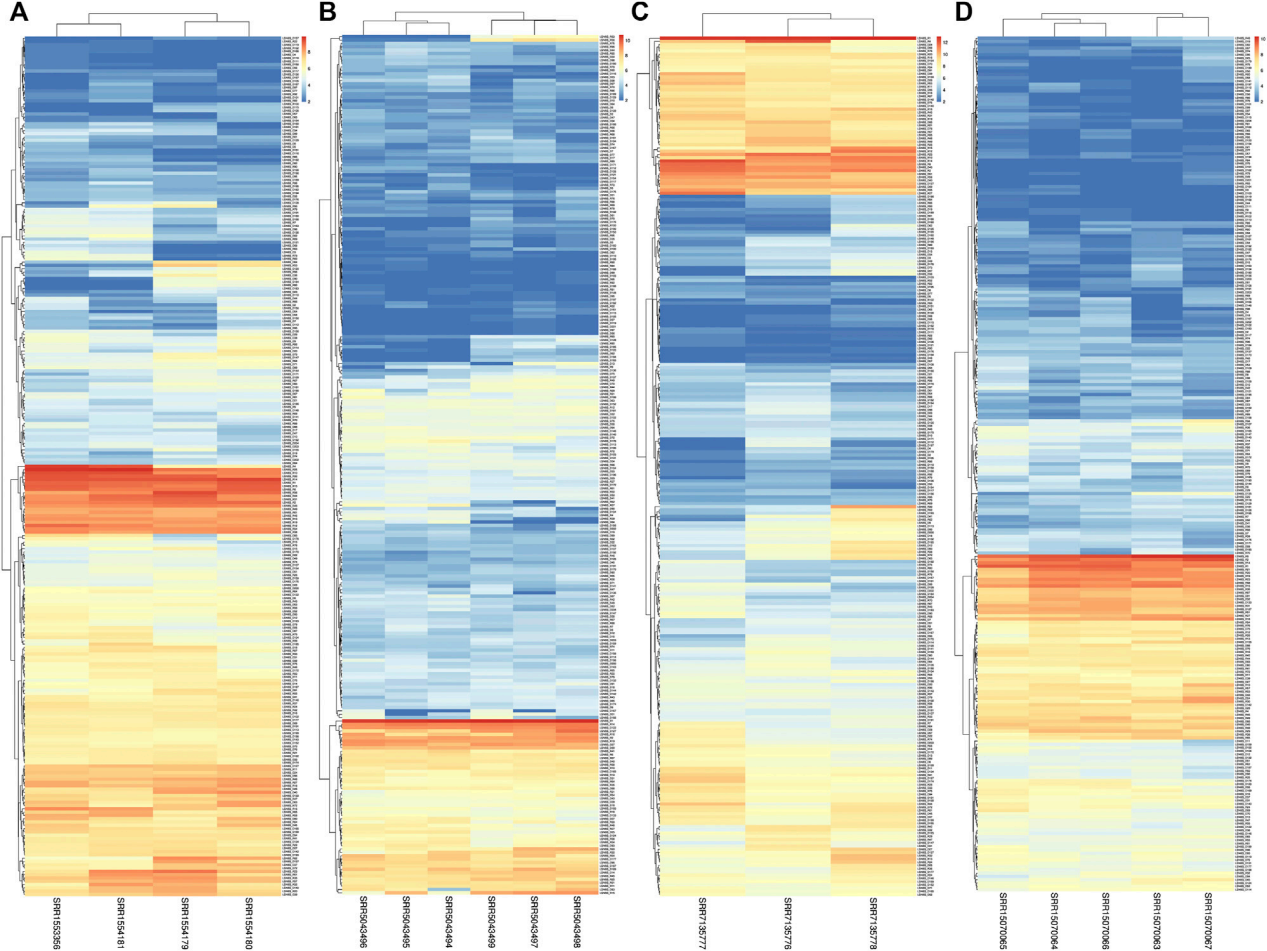
The heatmaps show the expression levels of LsNBS genes in grass pea, as determined by normalized RNA-Seq counts from four different gene expression studies **(A)**: SRP045652, **(B)** SRP092875, **(C)** SRP145030, and **(D)** SRP327502). The color gradient ranges from blue (low expression) to red (high expression), with intermediate colors indicating moderate expression levels.

In this study, we utilized RNA-seq analysis to investigate the expression of LsNBSs under various stresses in grass pea. Gene expression analysis is a powerful tool for identifying novel transcripts and assessing gene activity (Wang et al., 2012). Our results revealed that all 274 LsNBS genes exhibited consistent transcriptome activity, with 85% of the genes showing high expression levels across all experiments, particularly in the SRP045652 experiment where the expression of LsNBSs was examined in seedlings subjected to fungal infection. Several genes, including *LsNBS-R18*, *LsNBS-D88*, *LsNBS-D142*, *LsNBS-D146*, *LsNBS-D180*, *LsNBS-D204*, *LsNBS-R4*, *LsNBS-R21*, and *LsNBS-R98*, showed a marked increase in expression levels under stress conditions. Furthermore, in the SRP145030 experiment, we observed a gradual increase in transcriptomic levels across plant maturation, with some genes exhibiting high expression levels, such as *LsNBS-D75*, *LsNBS-D43*, and *LsNBS-D40*. We have presented these results in a heatmap (Figure 6). Overall, our RNA-seq analysis revealed that the discovered R-genes in grass pea exhibit noticeable activity both under normal plant conditions and during plant stress.

### 3.6 Expression profiles of NBS-LRR genes under salinity stress

**FIGURE 7:**
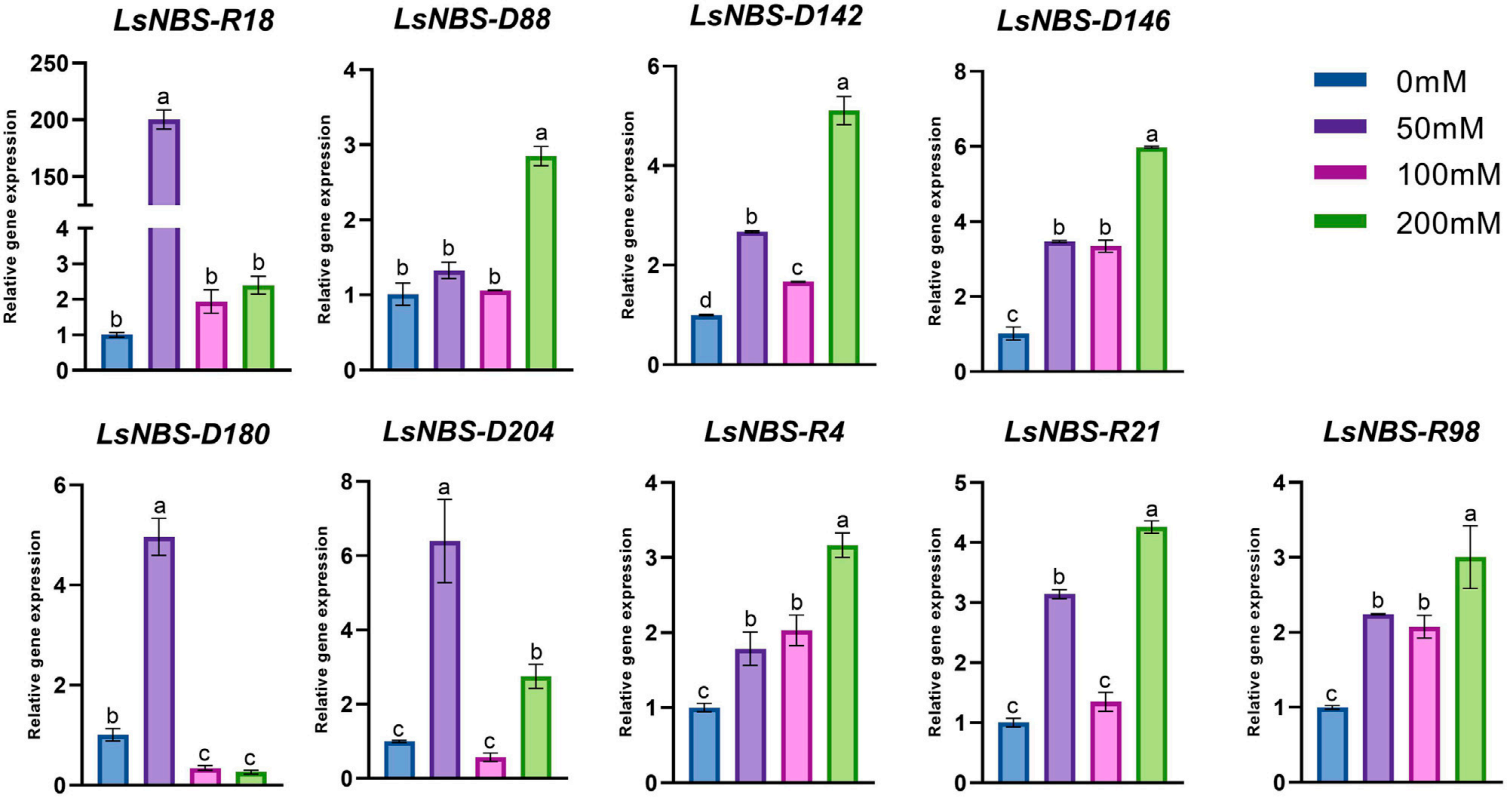
Expression patterns of the nine NBS-LRR-selected genes under salt stress. Where the expression levels of most NBS-LRR genes were upregulated at 50 and 200 *μ*M NaCl. Different letters in the graphs indicate means significant difference at P < 0.05 level between the treatments when compared with the control (Duncan’s multiple range test) and error bars represent SD.

We conducted gene expression validation using qPCR as a means to investigate the functions of potential gene family members of LsNBSs. Nine LsNBSs were chosen based on their clustering in the phylogenetic analysis and their expression levels in the RNA-seq analysis. We tried to select genes from different clusters in order to obtain a wide understanding of the gene family. Their expression profiles were screened under different levels of salt stress conditions. Our results, presented in Figure 7, indicate that the majority of the selected NBS-LRR genes exhibited expression patterns that agreed with the *in silico* functional analysis. In response to salt treatment, most NBS-LRR genes showed upregulation at 50 and 200 *μ*M NaCl. However, the expression patterns of *LsNBS-D18* and *LsNBS-D204* were reduced by 2.4-fold and 2.8-fold, respectively, compared to their respective 200-fold and 6-fold expression levels at 50 *μ*M NaCl treatment. Moreover, *LsNBS-D180* showed a drastic downregulation compared to its 5-fold up-regulation at 50 *μ*M NaCl. Overall, our gene expression validation results provide further insights into the potential functions of LsNBSs under salt stress conditions.

## 4 Discussion

Plants are constantly exposed to various pathogens and environmental stresses throughout their developmental stages, which has led to the evolution of a diverse defense system activated by resistance (R) genes (Yuan et al., 2011; Zhang W. et al., 2022). One of the largest R gene families in plants is the NBS LRR gene family, which encodes proteins containing nucleotide-binding sites (NBS) and leucine-rich repeat (LRR) domains that play a crucial role in detecting pathogenic infection signals (Yue et al., 2012; Zhang W. et al., 2022; Yin et al., 2023). Over the past few years, bioinformatics analysis has become an effective tool for identifying various gene families, including the NBS LRR gene family (Yang et al., 2022). The NBS-gene family has been investigated for the first time in the newly sequenced genome of grass pea (Emmrich et al., 2020). In this study, we characterized, annotated, and validated 274 potential NBS-encoding genes. Comparing the number of NBS genes in Lathyrus to other legumes, we noticed that there were 228 coding genes in beans (Wu et al., 2017) and 104 in chickpea (Sharma et al., 2017). Although LsNBS is comparable to Brassica rapa (206 genes), gene ratios differ slightly from *Brassica oleracea* (157 genes) and *Arabidopsis thaliana* (167 genes) (Yu et al., 2014). The high abundance of NBS-LRR genes in Lathyrus is likely due to the plant’s natural adaptation to its environment, which is characterized by pronounced environmental stresses. However, it is important to note that the frequency of NBS-LRR genes can vary greatly between different plant species, and studies have shown that there is no clear association between the frequency of NBS-LRR genes and either genome size or total number of annotated genes.

The identification of evolutionary linkages between grass pea and other legumes using NBS gene families would be a significant step forward in understanding its emergence and evolution. Based on phylogenetic analysis, the 274 LsNBS-encoding genes were classified into the TNL and CNL subfamilies. Among these, the TNL subfamily included 124 genes, while the remaining 150 genes belonged to the CNL subfamily. The CNL:TNL gene ratio was approximately 75:62, with the number of CNL genes slightly larger than the number of TNL genes (Figure 1). This LsNBS ratio is comparable to that of other legumes such as beans and chickpeas, which have a ratio of 34:39 (Sagi et al., 2017). Notably, in contrast to Brassicaceae species, where the TIR domain is more functionally active than the CC domain, the abundance patterns of TNL and CNL genes in *Lathyrus sativus* indicated that both TIR and CC domains have equivalent functional activity (Ma et al., 2021).

The TIR and CC domains exhibit clear structural and functional differences. The TIR domain adopts a flavodoxin-like fold consisting of a central five-stranded parallel *β*-sheet (strands *β*A–*β*E) flanked by five *α*-helices (*α*A–*α*E) on both sides (Ve et al., 2015), whereas CC domains are mainly composed of helical proteins (Bentham et al., 2018). Despite their roles in signaling and resistance specificity, the associated pathways of TIR and CC domains are distinct. The TIR domain primarily engages in self-association and homotypic interactions with other TIR domains (Bernoux et al., 2011; Ve et al., 2015; Ma et al., 2021). In plants, a single genome typically encodes numerous NBS-LRR genes, which are vital for protecting against a wide range of rapidly evolving pathogens (Ma et al., 2021). According to reports, dicots possess a higher number of CNLs than TNLs. Moreover, the TIR motif is uncommon in disease resistance genes in monocots such as *O. sativa*, *T. aestivum*, *Z. mays*, and *B. distachyon* (Zhou et al., 2004; Gu et al., 2015; Singh et al., 2015). This observation could explain why grass pea, a legume dicot, has a greater number of CNLs (150) than TNLs (124). This finding is consistent with other dicots such as *V. vinifera* (203 CNLs and 97 TNLs), *M. esculenta* (117 CNLs and 29 TNLs), and *S. lycopersicum* (117 CNLs and 29 TNLs) Andolfo et al. (2014).

We investigated the exon-intron structure and distribution among LsNBSs, providing valuable evidence for their evolutionary relationships. The gene structure supported the classification of the phylogenetic analysis and revealed a clear perspective on gene clustering based on their domain similarities and differences (Figure 2). The identified ten major motifs in the structure of LsNBS genes provide further evidence for their evolutionary relationships and functional significance. Interestingly, the number and type of motifs were found to be comparable to those of beans, *A. thaliana* (Meyers et al., 2003), *P. trichocarpa* (Kohler et al., 2008), *Dioscorea rotundata* (Zhang et al., 2020) and Chickpea (Sagi et al., 2017; Mokhtar et al., 2023b). The major motifs include P-loop, Walker_B, Kinase-GTPase, ABC, ChvD, Walker_B, Rnase H, Smc, and CDC48. The ChvD protein, which is an ATP-binding transporter homologous protein, is an example of unreported motifs that are associated with ATP-binding regulation Liu et al. (2001). Analysis of LsNBS motifs revealed their direct involvement in pathogen resistance and the ability to withstand harsh conditions, possibly explaining the absence of other motifs such as GLPL and MHDV, which have similar functions (Xu et al., 2005). Additionally, an N-terminal TIR/CC domain, a central NBS domain, and a C-terminal LRR domain are the three domains that makeup NBS-LRR genes (Song and Nan, 2014). TIR-1 and -2 were discovered at the N-terminus of the identified grass pea LsNBS-TNL genes (Figure 2), some of which were founded in the N-terminus of TNL genes identified in *P. trichocarpa*, *A. thaliana*, and common bean (Meyers et al., 2003; Kohler et al., 2008; Wu et al., 2017).

Protein interactions play a crucial role in the activity of proteins and form the basis for the molecular networks that regulate biological processes in organisms (de Folter et al., 2005). The study demonstrated a significant relationship among LsNBSs in performing their roles, where interaction relationships were observed between some of the identified LsNBSs. The low interaction observed with other LsNBSs could be due to the lack of information published in databases and literature. These results highlight the need for further studies on NBS in legumes to uncover possible correlations between genes responsible for critical biological functions such as disease resistance. The gene enrichment analysis confirmed the important role of LsNBSs in plant defense response. Several genes associated with responses to stimuli, defense, and other functions related to cell defense against infections were identified, supporting the involvement of LsNBSs in the immune system of *Lathyrus sativus*. Functional annotation revealed that LsNBS genes are involved in ATP binding, ADP binding, cyclic ADP-ribose generation, and ubiquitin binding, highlighting the ligand-interacting nature of highly conserved LsNBS domains. Furthermore, at the cellular component level, some LsNBSs are involved in cellular anatomical entities and the plasma membrane (Supplementary Table S9). These findings provide a better understanding of the molecular mechanisms underlying their importance in plant defense against pathogens. Additionally, it could serve as a guide for targeting NBS genes involved in plant defense against particular diseases. For instance, in *Phaseolus vulgaris*, NBS genes were used to magnify the search for genes associated with anthracnose resistance. This strategy involved identifying typical resistance proteins located near resistance loci in the common bean reference genome. By checking for proteins with NBS-LRR domains and kinase domains, researchers were able to pinpoint potential candidate genes for anthracnose resistance (Vaz Bisneta and Gonçalves-Vidigal, 2020).

The unique interactions between *cis*-acting elements and transcription factors located in gene promoters play a crucial role in controlling target gene expression (Zhao Y. et al., 2020). When plants are exposed to pathogenic infections, transcription factors are recruited by *cis*-acting sites in promoters, thereby initiating the transcription of resistance genes (Wang et al., 2020). Given the challenging conditions of climate and infections that grass pea is exposed to, it is not surprising that LsNBS genes have 103 different *cis*-elements (Figure 5), including four different types of hormone-responsive regulatory elements: salicylic acid responsiveness, MeJA responsive element, Ethylene Responsive Element, and abscisic acid responsiveness. The presence of many stress-sensitive proteins, such as WUN and W-box, should also be noted (Supplementary Table S8). This research is consistent with previous findings in *Vitis vinifera* during powdery mildew infection (Goyal et al., 2020) and resembles the NBS gene family found in Kabuli and common bean (Sharma et al., 2017; Wu et al., 2017).

In our study, we employed two different methods to validate the gene expression of the reported LsNBSs. Firstly, we used previous gene expression data for RNA-seq analysis. This analysis enabled us to examine the transcript levels of the LsNBS genes, revealing that nearly 230 LsNBSs have shared expression across all studied experiments. These genes were particularly highly expressed in SRP045652, which represents the primary function of NBS-LRR genes in disease resistance against fungal infections compared to other experiments. These findings suggest that some of the reported LsNBS genes could play a potential role in pathogen resistance (Figure 6). Secondly, we employed qPCR as another method to validate the expression of reported LsNBS genes. The majority of up- or downregulated NBS-LRR genes have been shown to confer resistance against various plant pathogens (Lv et al., 2022; Qian et al., 2022; Zhou et al., 2022). However, only a few NBS-LRR-encoding genes are responsive to abiotic stresses (De Leon et al., 2016; Subudhi et al., 2020; Filippou et al., 2021). Thus, investigating the response of NBS-LRR genes at different salinity stress levels would be intriguing. CNL and TNL are well-known plant sensors involved in biotic stress perception and signaling, and their roles in salt stress conditions are less understood. The fact that CNL (*LsNBS-D18*, *LsNBS-D88*, *LsNBS-D142*, *LsNBS-D180*, *LsNBS-D204*, *LsNBS-R4*, and *LsNBS-R98*) and TNL (*LsNBS-D146* and *LsNBS-R21*)-selected genes in grass pea respond to salt stress suggests their potential over-expression as a strategy to mitigate the negative impacts of salt stress. Our gene expression validation further unraveled the distinct adaptive strategies adopted by NBS-LRR genes during co-evolution with stressful environments, facilitating the integration of environmental cues into complex transcriptional responses (Figure 7). Future research will be required to determine the applicability and precise mechanisms of these intriguing players.

## Data Availability

The datasets presented in this study can be found in online repositories. The names of the repository/repositories and accession number(s) can be found in the article/Supplementary Material.
